# Human Activity Recognition: Review, Taxonomy and Open Challenges

**DOI:** 10.3390/s22176463

**Published:** 2022-08-27

**Authors:** Muhammad Haseeb Arshad, Muhammad Bilal, Abdullah Gani

**Affiliations:** 1 Department of Computer Science, National University of Computer and Emerging Sciences, Chiniot-Faisalabad Campus, Chiniot 35400, Pakistan; 2 Department of Software Engineering, National University of Computer and Emerging Sciences, Chiniot-Faisalabad Campus, Chiniot 35400, Pakistan; 3 Faculty of Computing and Informatics, University Malaysia Sabah, Kota Kinabalu 88400, Sabah, Malaysia

**Keywords:** human activity recognition, computer vision, CCTV, sensors, machine learning

## Abstract

Nowadays, Human Activity Recognition (HAR) is being widely used in a variety of domains, and vision and sensor-based data enable cutting-edge technologies to detect, recognize, and monitor human activities. Several reviews and surveys on HAR have already been published, but due to the constantly growing literature, the status of HAR literature needed to be updated. Hence, this review aims to provide insights on the current state of the literature on HAR published since 2018. The ninety-five articles reviewed in this study are classified to highlight application areas, data sources, techniques, and open research challenges in HAR. The majority of existing research appears to have concentrated on daily living activities, followed by user activities based on individual and group-based activities. However, there is little literature on detecting real-time activities such as suspicious activity, surveillance, and healthcare. A major portion of existing studies has used Closed-Circuit Television (CCTV) videos and Mobile Sensors data. Convolutional Neural Network (CNN), Long short-term memory (LSTM), and Support Vector Machine (SVM) are the most prominent techniques in the literature reviewed that are being utilized for the task of HAR. Lastly, the limitations and open challenges that needed to be addressed are discussed.

## 1. Introduction

Humans engage in a wide range of activities in their daily lives. The recent advancement in technology and data from Closed-Circuit Television (CCTV) and sensors has enabled the detection of anomalies as well as the recognition of daily human activities for surveillance [1,2]. The term anomaly refers to abnormal or unusual behavior or activity [3]. Human Activity Recognition (HAR) has been treated as a typical classification problem in computer vision and pattern recognition, to recognize various human activities [4]. HAR based on visual and sensory data has a huge number of potential applications and has piqued the interest of researchers due to rising demand. There is also an ongoing debate about the effectiveness of sensor-based HAR techniques versus vision-based HAR techniques. Currently, HAR has been utilized in diverse application domains including healthcare, surveillance, sports and event analysis, elderly care, and Human-Computer Interaction (HCI) [4]. The accuracy of HAR depends on a number of factors such as lighting, background, crowded scenes, camera viewpoint, and action complexity [5]. The widespread use of HAR applications has significantly improved human safety and well-being all over the world [6].

Anomalies are variously referred to as abnormalities, deviants, outliers, and unusualness in the literature. In real-time, an intelligent video surveillance system detects anomalies and anomalous entities like weapons in sensitive areas and abandoned objects. The video contains anomalies that are ambiguous, novel, unknown, uncommon (rare), irregular, unexpected, typical, and non-dictionary in nature [7]. Automatic crowd analysis assists humans in detecting threats and anomalous events by analyzing crowd modeling, crowd tracking, density estimation, and counting, and crowd behavior understanding. The general flow includes monitoring activities, identifying features, and detecting irregular activity [8]. Sensors, wireless communications, and machine learning algorithms have enabled the development of new systems with medical and assistive technologies that have provided an age-friendly environment and improved the life quality of older people. Sensor-based HAR learns activities through a series of observations and consists of five steps: sensor selection, data collection, feature extraction, model training, and model testing [9]. Abnormal behavior is difficult to define because it varies depending on the situation, but detecting it is critical. Individual-based and holistic approaches are used to detect unusual behavior in a crowd [10].

In recent years a number of systematic reviews and survey papers have been published for HAR [5,6]. However, the majority of them focused on specific tasks such as crowd surveillance [8], fall detection [3], healthcare [9,11], etc. Moreover, few of them targeted particular data sources such as sensors-based data for HAR [4,6,12]. Hence, this review aims to provide insights on the status of overall research work done in the HAR domain irrespective of task and data source. The four research contributions made in this review paper are as follows. First, a taxonomy of HAR is generated based on recent literature reviewed in Section 3, and the studies are organized based on the application areas in Section 4. Second, insights on techniques used for HAR used by existing literature are provided in Section 5. Third, the key data sources that are being used in the studies reviewed are identified and reported based on their types in Section 6. Finally, Section 7 highlights and discusses open research challenges that need to be considered and addressed by future studies while Section 8 discusses opinions on existing HAR studies.

## 2. Materials and Methods

**Figure 1:**
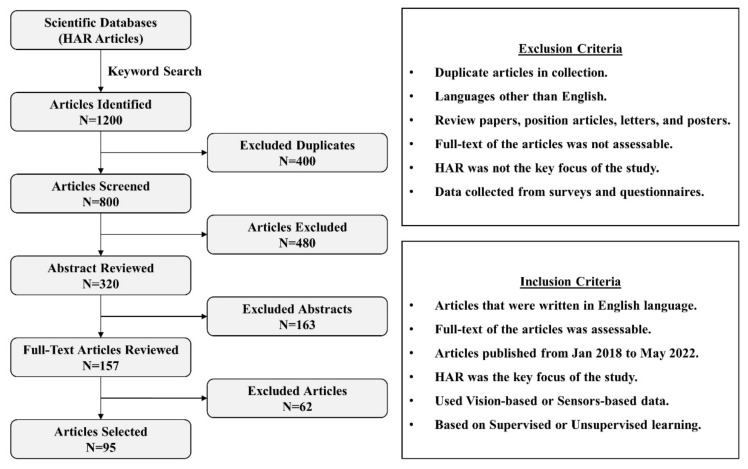
Steps performed for selection of articles.

HAR research articles published between January 2018 and May 2022 were collected using IEEE Xplore, SpringerLink, ACM, Google Scholar, and ScienceDirect. Other databases were also looked into, but access to them was limited. “Action Recognition”, “Action Detection”, “Activity Detection”, “Suspicious Activity Detection”, “Human Object Interaction”, “Multiple Actor Activity”, “Object Detection”, “Multi-Human Action Detection”, “Continuous Activity Recognition”, “Group Behavior Analysis”, “Abnormal Behavior Recognition”, “Violent Event Analysis”, “Event Detection”, and “Behavior Detection” were used as the keywords. Figure 1 shows the flow of steps performed for the selection of articles in this study. Initially, 1200 articles were collected using a keyword search, of which 95 were chosen for review. The articles were excluded in four steps. First, the duplicate articles were removed. Second, articles written in languages other than English, articles with inaccessible full-text, and position articles, letters, and posters were all excluded. Third, based on the abstracts, articles not meeting the screening criteria were removed. Fourth, articles not meeting the screening criteria were removed after a full-text review. The articles were collected over a thirty-day period in January 2022. Duplicates were removed, and preliminary screening was completed in February 2022. Abstracts were read in March 2022, followed by full-text screening and tabulation of included articles in April and May 2022. Another week in June 2022 was spent looking for articles published up to May 2022. This article was written from June to July 2022.

## 3. Human Activity

Human activity is defined as the sequential action of one or more people. HAR is the task of automatic detection and identification of human activities using various state-of-the-art techniques [5]. The activities can be indoors, such as sitting, lying down and walking as well as outdoor activities such as playing football or horse riding. In the literature, HAR is being used for different application areas [6]. This study organized existing literature on HAR under three categories including daily living, real-time and user activities. The daily living activities are further classified into static activities such as standing or sitting and dynamic activities such as walking or running. The studies that explored real-time activities are grouped under healthcare, suspicious and surveillance. Finally, the studies related to user activities are grouped into individual and group activities. It is interesting to note that majority of existing studies reviewed in this study are classified under daily living and user activities, while very limited studies are related to real-time activities.

The nature and availability of data has a vital role in HAR. The data being used by researchers for HAR is of different types and comes from vision-based and sensors-based data sources. This study grouped existing literature based on the type of data such as video, image, and sensor data. Despite the discussions on the advantages of sensors-based data over vision-based data, the majority of existing studies rely on vision-based data for HAR. The vision-based data is further classified based on the type of data such as video or image. The videos being used in HAR literature are collected from CCTV, smartphones, Kinect device and YouTube, while on the other hand social media and camera images are used for vision based HAR. Mobile sensors and wearable body sensors are two types of sensors-based data sources used in existing literature.

HAR has been studied from the perspective of both supervised and unsupervised classification problems in the existing literature. A wide range of various traditional machine learning and cutting-edge deep learning algorithms used by researchers for HAR are highlighted. It is seen that majority of the existing literature took HAR as a supervised classification in contrast to unsupervised classification. The most prominent supervised techniques used for HAR includes Convolutional Neural Network (CNN), Long short-term memory (LSTM), and Support Vector Machine (SVM). SVM is a traditional machine learning algorithm which is preferred for small sized datasets, while CNN and LSTM are state-of-the-art deep learning techniques that requires larger datasets.

**Figure 2:**
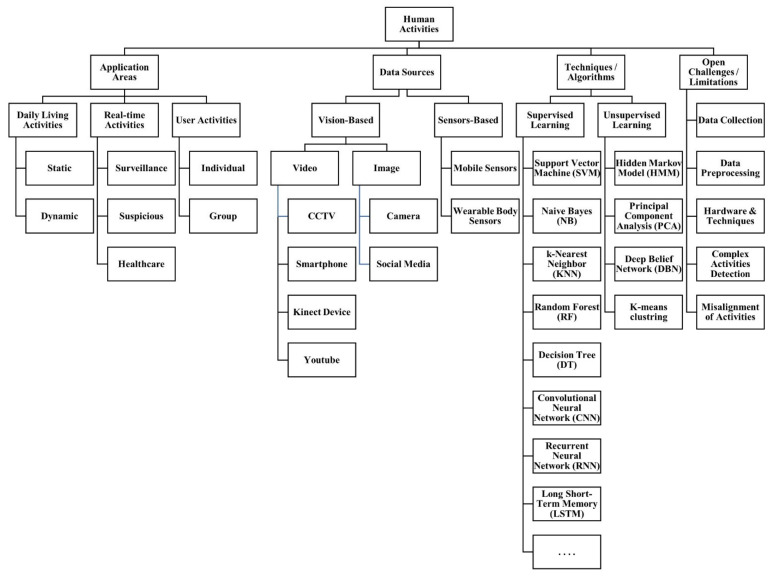
Taxonomy of HAR.

The open challenges and limitations of existing HAR studies are listed under five categories including data collection, data preprocessing, hardware and techniques, complex activity detection and misalignment of activities. Vision-based data is of bigger size and requires more processing compared to sensor-based data. However, the cost of sensors is comparatively much higher than vision-based data capturing devices. The overall taxonomy of existing literature on HAR created is shown in Figure 2.

## 4. Application Areas

**Figure 3:**
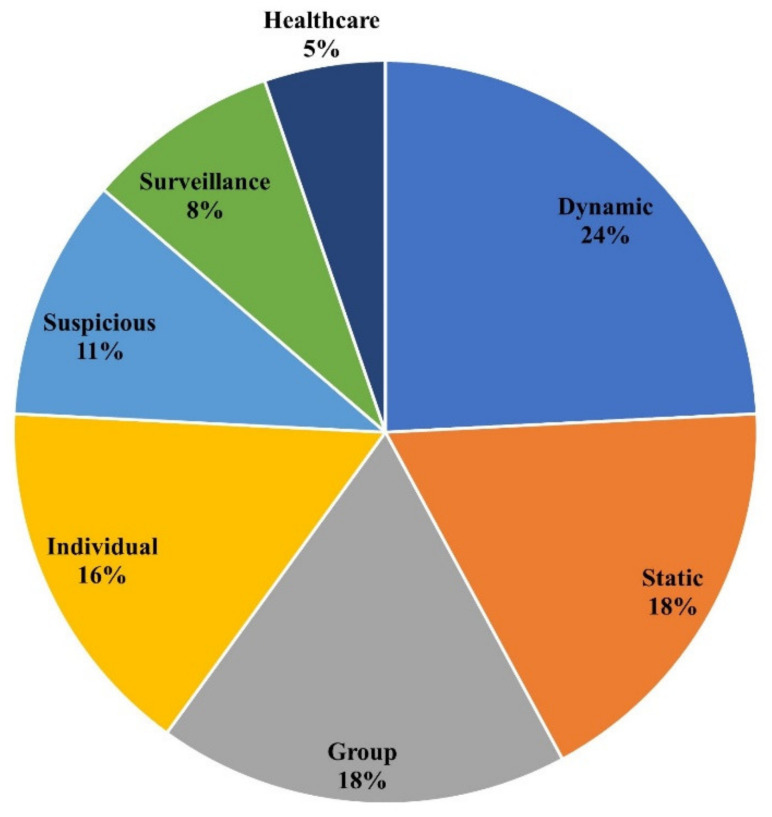
Frequency of application areas targeted by existing literature on HAR.

As noted earlier, this study grouped existing HAR literature based on different types of activities including daily living, real-time, individual, and group-based activities. Each category is discussed in detail in the related sections below. Figure 3 shows the frequency of literature on HAR based on articles reviewed in this study.

### 4.1. Daily Living Activities

Daily living activities can be seen from two perspectives: static and dynamic. Static activities are those in which an individual is fixed with respect to an observer such as a camera or sensors, whereas dynamic activities involve the individual moving consistently. Researchers have presented a variety of HAR solutions in the literature.

#### 4.1.1. Static Activities

Köping et al. [13] proposed a framework based on SVM to collect and store data on a central server using a smartphone and eight different sensors. Afterwards, the data is encoded into a feature vector. Their experiments showed that the proposed framework detects real-time static and dynamic activities, with an accuracy of 87.1%. Different extracted features (mean, median, autoregressive coefficients) using a smartphone inertial sensor refined by Kernel Principal Component Analysis (KPCA) and Linear Discriminant Analysis (LDA) make them vigorous. Hassan et al. [14] introduced a Deep Belief Network (DBN) that trained those attributes for HAR. It was seen that DBN outperformed SVM and Artificial Neural Network (ANN) for HAR. Sukor et al. [15] used Principal Component Analysis (PCA) to extract the most relevant features from a mobile phone’s tri-axial accelerometer and gyroscope sensor data in the form of signals. Experimental results showed that the proposed algorithm achieved 96.11% accuracy compared to other machine learning classifiers on a publicly available dataset.

Bota et al. [16] introduced a Semi-Supervised Active Learning (SSAL) approach to relatively automate the annotation process for HAR based on Self-Training (ST). SSAL reduces the annotation effort to produce the required volume of annotated data to obtain the best classifier. The researchers compared supervised and unsupervised methods on University of California Irvine (UCI) (https://archive.ics.uci.edu/ml/datasets.php (accessed on 16 July 2022)) datasets and showed an 89% possibility of reduction. Zhu et al. [17] proposed a semi-supervised deep learning approach to implement temporal ensembling of deep long-short term memory (DLSTM) on labeled as well as unlabeled data. Smartphone inertial sensors utilized to collect data and its characteristics were obtained with deep neural network (DNN) for local dependencies. Researchers compared their results with several algorithms evaluated on the UCI dataset to produce state-of-the-art results. Du et al. [18] proposed a framework that uses RFID tags to recognize and predict in advance human activity, together with post-activity recognition, and recognition in progress. The smart home can play an important role in health care, power saving, etc., and enables the operation of smart services according to the human mind. Experimental results on two residents performing daily living activities showed that the recognition precision can reach 85.0% and the prediction accuracy is 78.3%, which is higher in terms of accuracy as compared to Naive Bayes on the Ordonez dataset. Machine learning, IoT, and powerful computers have improved the performance of smart spaces. To determine static and dynamic actions, the Shelke and Aksanli approach [19] represents a low-cost, low-energy smart spaces implementation. On data collected with low-resolution (4 × 16) thermal sensors, the researcher trained LR, NB, SVM, DT, RF, and ANN (vanilla feed-forward). According to the experimental results, ANN achieved 99.96% accuracy on continuous HAR.

Chelli and Patzold [20] developed a system that recognizes seven different activities, including fall detection. A mobile phone was used to extract time and frequency domain features from acceleration and angular velocity. The system achieved 81.2% accuracy with ANN, 87.8% for K-nearest neighbor (KNN), 93.2% for Quadratic SVM (QSVM), and 94.1 percent accuracy in ensemble bagged tree (EBT) on acceleration data only. Extracted features from acceleration and angular velocity expand the accuracy by 85.8%, 91.8%, 96.1%, and 97.7% for all the above-mentioned algorithms, and the accuracy of QSVM and EBT for fall detection reaches 100% without any false alarm which is the best possible performance. K. Chen et al. [21] addressed both the limitations of labelled data and the challenges of multimodal sensor data by introducing a pattern-based semi-supervised deep recurrent convolutional attention network framework (RCAM) with wearable sensors to handle the imbalanced distribution of labelled data. Experimental results demonstrated the proposed method’s robustness over imbalanced and small training datasets. Javed et al. [22] proposed a Multilayer Perceptron (MLP) classifier to predict physical activities with data collected using a smartphone accelerometer sensor with two axes from 12 participants performing daily living activities. While testing on a publicly available WISDM data set, MLP achieved 93% weighted accuracy, which is nearly 13% higher than existing methods.

Tong et al. [23] investigated that HAR progress has stalled because sensors do not provide enough information to recognize activities. Image sensing hardware and modeling techniques do not burden modern mobile phone hardware and will open many opportunities for activity recognition in the future. Ahmed et al. [24] proposed a hybrid method feature selection process to work efficiently with limited hardware. The process includes a filter and a wrapper method. A study used sequential floating forward search (SFFS) to extract anticipated features and SVM to classify daily living human activities. SFFS overcomes the sensor’s high dimensionality problem with 96.7% accuracy. The unavailability of labeled data, higher computational costs, and system resource requirements are issues associated with HAR. Khan and Ahmad [25] proposed an attention-based multi-head model with three one-dimensional convolutional heads to address these issues and achieved state-of-the-art results on the WISDM and UCI HAR datasets. Tri-axial gyroscope and tri-axial accelerometer of wearable devices and the internet of things (IoT) are used to obtain advanced information about human behavior and can be considered biometric qualities. Biometric qualities were used for identifying people using deep learning namely CNN and LSTM. Experimental results showed the highest accuracy of 91.77% for CNN and 92.43% for LSTM [26]. In a series of experiments on the USC-HAD (https://www.cis.fordham.edu/wisdm/dataset.php (accessed on 16 July 2022)) dataset, Mobiact (https://bmi.hmu.gr/the-mobifall-and-mobiact-datasets-2/ (accessed on 16 July 2022)), Motionsense (https://www.kaggle.com/datasets/malekzadeh/motionsense-dataset (accessed on 16 July 2022)), and UCI-HAR datasets, Haresamudram et al. [27] focused on the collection of unlabeled data using mobile phone sensors. With the effective use of labeled data, human activities can be recognized using the Contrastive Predictive Coding (CPC) framework, which leads to improved recognition performance.

Traditional NN and deep learning techniques have made significant advances in many areas of life, including healthcare. Some existing techniques have shortcomings such as ignoring data variability, having a large number of parameters, consuming a large amount of resources, and being difficult to implement in real-time embedded devices. Pan et al. [28] aimed to address these issues by employing the GRU network, which collects valuable moments and temporal attention in order to minimize model attributes for HAR in the absence of independent identical distribution (I.I.D.). GRU and time focus the proposed method which, according to the researchers, outperforms existing technologies in terms of the aforementioned characteristics and can be implemented in low-cost embedded machines. Nowadays, a mobile phone is an indispensable part of everyday life. Its computational power, storage capacity, and sensor quality are all improving. Many existing studies have used it in conjunction with various techniques for various purposes, such as HAR. Luwe et al. [29] proposed a hybrid model that combines one-dimensional CNN with bidirectional LSTM (1D-CNN-BiLSTM) to recognize individual actions using wearable sensors. 1D-CNN converts visible features gathered by sensors to indicative features, while BiLSTM encodes broad dependencies through a gating process. The proposed method outperformed existing methodologies, achieving 95.48% recognition accuracy on the UCI-HAR dataset. The summary of the literature related to static activities is given in Table 1.

#### 4.1.2. Dynamic Activities

Saini et al. [31] proposed a framework based on a Bidirectional Long Short-Term Memory Neural Network (BLSTM-NN) to capture 3D skeleton trajectories for recognition of 24 continuous human activities using Microsoft Kinect. The results showed 68.9% accuracy for sitting positions without length modeling. Rueda et al. [32] proposed a novel deep neural network to recognize static and dynamic activities from a sequence of multichannel time-series signals acquired from various body-worn devices. Researchers achieved the best results on Opportunity (https://archive.ics.uci.edu/ml/datasets/opportunity+activity+recognition (accessed on 16 July 2022)), Pamap2 (https://archive.ics.uci.edu/ml/datasets/pamap2+physical+activity+monitoring (accessed on 16 July 2022)), and an industrial dataset. Begin and end labels of activity can identify sensor-based activity in the dataset with fixed window size, but it is not possible to foresee the start time of activity. Espinilla et al. [33] proposed online activity recognition to recognize daily living activities such as showering, toilet, eating, waking up, and so on with three temporal sub-windows that use only the end time of activity. Experimental results showed temporal sub-window improved the accuracy to 98.95% on the VanKasteren, Ordonez (https://deeplearning.buzz/deep-learning-datasets/ (accessed on 16 July 2022)) dataset.

Qi et al. [34] proposed a DCNN model for complex HAR using a smartphone. DCNN integrates several signal processing algorithms for data collected from three types of sensors. The proposed DCNN model computed quickly and had a high accuracy of 95.27% in evaluations on a dataset that contains twelve complex activities. Alghyaline [35] proposed an approach based on YOLO object detection, Kalman Filter, and Homography to detect real-time static and dynamic activities from CCTV camera videos with more than 32 fps. The results showed an accuracy of 96.9% for the BEHAVE (https://virtualhumans.mpi-inf.mpg.de/behave/ (accessed on 16 July 2022)) dataset and 88.4% for CCTV datasets.

Zhang et al. [36] used I3D to combine long-term activities and graph convolutional networks for interaction between actors and objects. I3D simplifies optimization and improves baseline by 5.5 percent mAP over 4.8 percent mAP evaluated on the AVA dataset. Chen et al. [37] proposed an Extreme Learning Machine (ELM) algorithm to classify and recognize human activities using smartphone sensors. Experimental results showed 97.35% and 98.88% accuracy on two public datasets. H. Ma et al. [38] proposed the AttnSense model to capture signal sensing dependencies with gyroscope and accelerometer sensors. A combination of CNN and a gated recurrent network (GRN) sense signals in spatial and temporal domains. Experimental results showed competitive performance in activity recognition on three publicly available datasets. Almaadeed et al. [39] extracted data into a new representation from each person performing multiple activities in the same surveillance video, which is then used to detect the corresponding action. They used multiple human action recognition using 3Dimensional deep learning trained on KTH, Weizmann, and UCF-ARG datasets. 3Dimensional deep learning achieved 98% accuracy as compared to other state-of-the-art methods on UCF101, Hollywood2, HDMB51, and YouTube.

Gleason et al. [40] proposed a two-stage approach for HAR and demonstrated its effectiveness on various types of videos. The first stage generated dense spatio-temporal proposals on frame-wise object detection using hierarchical clustering and jittering techniques. Action classification and temporal refinement in untrimmed videos were performed in the second stage using the Temporal Refinement I3D (TRI-3D) network. Statistical information-based strategies do not support activity recognition well because it is entirely dependent on the activity feature-solving strategy. TF-IDF based activity feature-solving strategies highlight statistical data of an individual’s activity. Three classifiers among multiple deep learning algorithms evaluated on tulum2009 and Cairo (https://knoema.com/atlas/Egypt/Cairo/datasets (accessed on 16 July 2022)) datasets achieved the best results in smart homes [41]. Wu et al. [42] presented AdaFrame which predicts which frame has to be observed next to reduce computational cost. AdaFrame includes LSTM as well as search frames that use overtime to see more frames at each timestamp and achieved 8.21 frames on FCVID and 8.65 frames on ActivityNet.

Accurate detection of body parts is essential for recognizing physical activities. Nadeem et al. [43] proposed a framework that combined body part and discriminant analysis, with features extracted as displacement parameters that represent body part positions and processed using maximum entropy. The Markov model for markerless human pose estimation and physical activity recognition achieved 90.91% accuracy for body part detection on the UCF dataset. Experimental results showed 89.09% accuracy for activity recognition on the UCF YouTube action dataset and 88.26% accuracy on the IM-DailyRGBEvents dataset. Online temporal action detection from untrimmed videos is a difficult task because there are multiple actions in a single frame, including background scenes, and only past and current information are available in the online setting. Due to intra-class dissimilarity in human activities, previous methods are unsuitable for this task. Yoon et al. [44] proposed an online action detection framework to deal with the insufficient information issue, which takes actions as ordered subclasses and controls a forthcoming frame generation in video gaming to achieve state-of-the-art results.

Ma et al. [45] used the Multivariate Gaussian Distribution (MGD) method to find the difference between time window and activity features, as well as the issue of time duration that leads to poor recognition. While conducting experiments on wheelchair users’ daily activities, MGD performed 15.3% better in static conditions, as well as 6.4% and 24.5% better on flat and disarranged floors, respectively. Change Point-based Activity Monitoring (CPAM) reduced energy consumption by 74.64% while recognizing and monitoring complicated and daily living actions performed routinely, using smartphone and smartwatch sensors. Experimental results evaluated on data from 66 subjects showed that the CPAM method is effective for reducing the energy footprint [46]. Pan et al. [47] developed Actor-Context-Actor Relation Network (ACAR-Net) that enables unintended relation reasoning for spatiotemporal action localization. Fine-grained action differences and multiple co-occurring interaction problems are addressed in various ways, but the result is a large combination of space and non-interactive pair dominance. Lin et al. [48] proposed Action-Guided Attention Mining and Relation Reasoning (AGRR) networks, which use contextual compatibility, consistency and class-activation map mining to identify human-object interaction. AGRR outperforms other approaches on images from the V-COCO and HICO-DET datasets.

Temporal action segmentation is divided and refined into framewise action classifications with the Action Segmentation Branch (ASB) and action boundary regression with Boundary Regression Branch (BRB). Ishikawa et al. [49] proposed the Action Segment Refinement Framework (ASRF) to improve performance on challenging datasets up to 13.7% in terms of segmental edit distance and 16.1% in terms of segmental F1 score. Grey wolf optimizer (GWO) improved the performance of the Gradient-based Optimizer (GBO) algorithm by selecting the appropriate features. Helmi et al. [50] used SVM to classify the activities because HAR plays an important role in every field of life. GBO achieved 98% accuracy on well-known publicly available UCI-HAR and WISDM datasets. Li et al. [51] proposed joint domain and semantic transfer learning (JDS-TL), which consists of 2 sections: unsupervised domain adaption and supervised semantic transfer. JDS-TL reduced the need for labeling a large number of radar signals. It is very difficult and time-consuming to obtain a handsome radar dataset with trustworthy labels, limiting deep learning models’ generalizability. A public radar micro-Doppler spectrogram dataset containing 6 human actions has an average accuracy of 87.6%, according to the experimental results.

HAR is becoming more common in a variety of industries, including healthcare. The researcher proposed an insole-based system to investigate the significance of data dissection, sensor use, and attribute selection. D’Arco et al. [52] used SVM to identify daily living activities in the proposed system by adjusting the size of the sliding window, reducing features, and implanting inertial and pressure sensors. The system’s accuracy was 94.66% while both sensors were used together. Inertial sensors, according to the findings, are best for dynamic actions, while pressure sensors are best for static actions. Implementing an appropriate and strong HAR system in real-world conditions is a significant challenge. Various researchers used pre-segmented sensor data to identify actions, but Najeh et al. [53] attempted to perform HAR using streaming sensors. They tried to figure out whether the currently performing action is a continuation of a previously performed activity or is novel in three steps: sensor correlation (SC), temporal correlation (TC), and determination of the activity activating the sensor. The experimental results on the real case study “Aruba” from the CASAS database revealed an F1 score of 0.63–0.99. The summary of the literature related to dynamic activities is given in Table 2.

### 4.2. Real-Time Activities

#### 4.2.1. Surveillance

To address real-time pedestrian detection issues, Jiang et al. [54] proposed an approach in which static sparse features are extracted using a fast feature pyramid, followed by sparse optical flow to obtain sparse dynamical features between frames. These two types of features are combined in Adaboost for classification. Experimental results showed yielding the best results on the TUD dataset. Basha et al. [55] proposed CNN-DBNN for automatic tracking and detection of criminal or brutal activities in videos. Discriminative Deep Belief Network (DDBN) receives features that are extracted from frames using CNN. Results showed an increase in accuracy of 90% for the proposed classification framework. R. [56] proposed a method to monitor traffic and unprecedented violence using CCTV cameras to identify the movement of objects, and synchronization provides object details. Researchers evaluated this proposed method in real time.

Actions are identified by comparing existing and generated Histogram of Oriented Gradients (HOG) of the frames of each shot’s Temporal Difference Map (TDMap). On a video dataset, the proposed CNN model for multiple action detection, recognition, and summarization (such as two people fighting, walking, hand waving, and so on) recognizes actions with an accuracy of 98.9% [57]. Qin et al. [58] proposed detecting and preventing criminal activities in shopping malls (DPCA-SM) using a video monitoring approach. DPCA-SM makes effective decisions such as tracing people’s routes and detecting measures of store settings in real-time using surveillance cameras and generating alerts. The proposed method was evaluated on real and private dataset CAVIAR and produced 92% accuracy in crowded conditions.

The introduction of machine learning, deep learning, and artificial intelligence to continuously monitor public spaces and intelligent video surveillance constitute advancements in technology. Human behavior is highly unpredictable and verification of suspicious or normal is difficult. The proposed framework is divided into two parts. The first section computes features from video frames, while the second predicts whether the computed features are suspicious. Mahdi and Jelwy [59] proposed an automated method for detecting unusual situations in academic situations and alerting the appropriate authority. The proposed system had a 95.3% accuracy rate. Video surveillance plays an important role in security measures, and the main goal of HAR is to reveal a variety of activities in videos. Sunil et al. [60] used a deep learning model to categorize and specify activities detected using a bounding box and the Single Shot Detector (SSD) algorithm. The model was tested in all classes and revealed real-time implementation issues. Argus++ is a vigorous real-time activity detection for evaluating unconfined video streams in the real world. To deal with multi-scale multi-instance cases and large fields-of-view with the help of cameras, it uses corresponding spatio-temporal cubes as the intermediate concept for action detection through over-sampling. Experimental results on different surveillance and driving scenarios on CVPR ActivityNet ActEV 2021, NIST ActEV SDL UF/KF, TRECVID ActEV 2020/2021, and ICCV ROAD 2021 demonstrated superior performance [61]. Table 3 shows the summary of the literature related to surveillance.

#### 4.2.2. Suspicious Activities

Mohan et al. [62] proposed a method for manually monitoring unusual anomaly activities in supermarkets, public places, and university campuses. PCA and CNN resolve the problem of manual procedures such as false alarms and reveal the location of an irregularity in the video. Frame-wise anomalous occurrence is detected by PCA and SVM classifier. The proposed method performed state-of-the-art results on UCSD, UMN dataset, and Avenue Dataset. Shoplifters can easily remove labels from products while being monitored by Electronic Article Surveillance (EAS) systems. Real-time video from CCTV cameras is sent to a CNN model to detect suspicious human activities in the store such as shoplifting, robbery, and break-in, and generate an alarm. The proposed system outperforms others with an accuracy of 89% [63].

Jyotsna and Amudha [64] used a deep learning approach with an accuracy of 87.15% to detect normal or abnormal activity from video frames in an academic environment, such as using a mobile phone on campus, walking, or fighting. This deep learning approach is composed of two parts, the first of which computes features from video frames and the second of which predicts suspicious or normal class. Khan et al. [65] proposed a framework that strategizes video statistics obtained from a CCTV digital camera fixed at a specific location. However, to detect suspicious human behavior in a large and complex area, several cameras must be set up at constant positions. A widget mounted in indoor environments triggers an alarm when unusual and suspicious moves are detected. Pyataeva and Eliseeva [66] proposed a method for detecting smoking events in visual data. The method, which works with video-based spatio-temporal features, employs the ResNet three-dimensional CNN. The proposed method recognized smoking actions with 15% greater accuracy than the other basic architectures on the HMDB51 dataset.

Riaz et al. [67] used a pre-trained model to extract features from videos for pose estimation. These features are then passed to a cascade of deep CNN to detect cheating in the exam hall using a camera. The proposed method achieved 95.88% accuracy on the video dataset. Mudgal et al. [68] proposed a smart and intelligent system to monitor normal and abnormal activities such as hitting, slapping, punching, and so on for real-time monitoring of sensitive locations such as airports, banks, and roads. The researcher combined the Gaussian Mixture Model (GMM) with the Universal Attribute Model and performed state-of-the-art feature vectors on the UCF101 human action dataset. Traditional security methods demand continuous human intervention. Deep learning is used to detect and warn users of potentially dangerous behavior. Amrutha et al. [69] tried to reduce the time and effort spent on video monitoring using deep learning and image processing. The proposed system attempted to detect real-world suspicious activities in surveillance videos, such as burglaries, assaults, and so on.

W. Ullah et al. [70] presented an efficient framework and a two-stream NN. The framework identifies abnormalities in surveillance Big Video Data using Artificial Intelligence of Things. The first stream consists of immediate anomaly detection with an IoT device. The second stream analyses anomalies by sending frames to the cloud analysis center, and the bi-directional long short-term memory (BD-LSTM) layer classifies anomaly classes. BD-LSTM reported a 9.88% and 4.01% increase in accuracy. Recent advances in video anomaly detection have only improved results on small datasets, but researchers are looking into practical challenges such as continuous learning and few-shot learning. Humans find these tasks simple, but machines find them extremely difficult. Doshi and Yilmaz [71] created an algorithm to deal with issues such as a vehicle driving in the wrong direction and a person loitering after midnight. The researcher tested his algorithm on a self-created dataset that was larger than existing datasets and other state-of-the-art datasets. The proposed algorithm outperformed others by a wide margin in both continuous learning and few-shot learning tasks. A summary of the literature related to suspicious activities is given in Table 4.

#### 4.2.3. Healthcare

Single sensing modality is a limitation in a smart healthcare environment. Gumaei et al. [72] proposed a robust multi-sensor-based framework employing a hybrid deep learning model to overcome this limitation. The framework contains simple recurrent units (SRUs) to process the sequence of multimodal data and gated recurrent units (GRUs) to store and learn from previous information. The proposed framework achieved more than 90% accuracy in less than 1.7 s of classification time on the MHEALTH dataset. Uddin and Hassan [73] proposed a Deep CNN that uses signals to extract features from various body sensors such as magnetometers, accelerometers, and gyroscopes. Deep CNN was established on Gaussian kernel-based PCA to recognize activities for smart healthcare. The approach is tested on the Mhealth dataset to determine its effectiveness and use for cognitive assistance.

Real-time monitoring can be performed by placing equipment such as wearable devices on the body of the person to recognize a particular feature such as falls, gait, and breathing disorders. However, these devices could be uncomfortable for a person who is being tracked all the time or may forget to wear them. Taylor et al. [74] demonstrated the detection of human motion with a quasi-real-time scenario using a non-invasive method. Taylor et al. [74] also produced a dataset of radio wave signals using software-defined radios (SDRs) to create test cases for standing up or sitting down and achieved 96.70% accuracy using the RF algorithm. Because medical images contain so much information about diseases, they can be used in real time to detect and meditate on various diseases. This makes a significant contribution to medical fields such as fitness tracking and elder care. Ref. [75] proposed a collection of models called “CNN-net,” “CNNLSTM-net,” “ConvLSTM-net,” and “StackedLSTM-net,” all of which are based on one-dimensional CNN and LSTM but differ in architecture. The researchers have named their proposed method Ensem-HAR. The above-mentioned classification techniques stacked predictions and then trained a blender on them for final prediction. The proposed model achieved 98.70%, 97.45%, and 95.05% accuracy on the WISDM, PAMAP2, and UCI-HAR datasets, respectively. The concept of the Internet of Healthcare Things (IoHT) can be applied to sensory data collected with mobile phone sensors for fall detection, smoking detection, healthcare, and other applications, but it is not limited to these applications. Ref. [76] used data collected with two smartphones to apply a model based on handcrafted features and RF. The results of the experiments show that the technique used outperforms others on the same data. On the WISDM v1 dataset, the applied method achieves 98.7% accuracy. The summary of literature reviewed related to the recognition of healthcare-related activities is given in Table 5.

### 4.3. User Activities

#### 4.3.1. Individual Activities

Human behavior is complicated and varies in both motion and appearance. Hsu et al. [77] used an unsupervised learning approach for a psychiatric patient using a camera, which includes an N-cut algorithm, an SVM, and a Condition Random Field (CRF) to label video segments. In smart surveillance, a unified framework based on a Deep convolutional framework is proposed by Ko and Sim [78] to detect abnormal human behavior from RGB images, such as punching, kicking, pushing, and so on, and provides satisfactory performance in a real-world scenario. It consists of three modules: one for separating object entities, one for extracting features from postures, and one for detecting abnormal behavior using LSTM.

The main challenge faced in the detection of HAR in live videos is the change in the territory or context of the scene. HOME FAST (Histogram of Orientation, Magnitude, and Entropy with Fast Accelerated Segment Test) spatiotemporal feature extraction approach based on optical flow can overcome these issues and identify the abnormal event in complex scenes under various transformations. HOME FAST can handle different anomalies and perform state-of-the-art results on UCSD, Live Videos (LV), and Avenue datasets [79]. Narrow areas and distortion caused by large depths of field can impede real-time violence detection. J. Zhang et al. [80] introduced an effective algorithm that uses an adaptive transformation mechanism and an improved pyramid L–K optical flow method to extract abnormal behavior features. The proposed algorithm improved the accuracy of abnormal behavior detection in narrow area scenes captured by CCTV cameras.

Custom-designed algorithms are tuned to detect only one specific type of behavior, but they may miss another type of behavior. Founta et al. [81] proposed a deep learning unified architecture that uses available metadata and combines hidden patterns to detect multiple abusive norms that are highly interrelated. Proposed architecture demonstrated 92% to 98% accuracy on Cyberbullying, Hateful, Offensive, Sarcasm, and Abusive datasets. Dou et al. [10] used SVM to determine and predict abnormal pedestrian behavior. SVM extracted feature vectors and vector trajectories of joint points determined by estimating the posture and optical flow field with a camera. Experimental results achieved 87.4% accuracy on the University of Minnesota’s abnormal population dataset. Moukafih et al., [82] proposed Long Short-Term Memory Fully Convolutional Network (LTSM-FCN) to improve traffic security. LTSM-FCN detects aggressive driving behavior and achieved 95.88% accuracy for a 5-min window length as compared to other deep learning models on the UAH-DriveSet dataset gathered by smartphone.

CNN extracts spatiotemporal features and handcrafted features such as Histogram of Optical Flow (HOF) and Histogram of Oriented Gradients (HOG) with Iterative Weighted non-Negative Matrix Factorization (IW-NMF). These features are combined for anomaly detection in the surveillance video sequence. Experimental results showed competitive performance on UCSD and UMN datasets as compared to other state-of-the-art methods [83]. It is extremely difficult to manually monitor CCTV video all of the time. Lee and Shin [84] proposed a deep learning model to detect abnormal behavior such as assault, theft, kidnapping, drunkenness, and so on. Experimental results showed I3D model is the most accurate among all others. Actions from images can be deduced by extracting 2D spatial features, but in the case of video, temporal information is required. Bhargava et al. [85] proposed an algorithm that can adapt to changing environments to do segmentation in bytes and prediction of anomalies in real-time video as technology advances.

Xia and Li [86] used a fully CNN, a pre-trained VGG-16 to extract static appearance features. The temporal attention mechanism extracts appearance features at the same position. LSTM network decoded these features to predict abnormal features in the moment to find abnormal behavior in video frames. The proposed method achieved the best results at the pixel and frame level when compared to others. Zhang et al. [87] proposed a method to extract global features, face alignment features, and facial action unit features. These features were provided as input to graph convolution for correlation and blended features classification action units based on multi-scale features. The proposed method achieved an accuracy of 0.674 on the Aff-Wild2 database.

Bhagya Jyothi and Vasudeva [88] proposed a Chronological Poor and Rich Tunicate Swarm Algorithm (CPRTSA)-based Deep Maxout Network to counteract the problem of changing human action. CPRTSA extracts effective features to recognize human activities in different domains such as intelligent video surveillance to improve security level. The proposed algorithm achieved 95.9 percent accuracy and 96.3 percent sensitivity. Belhadi et al. [89] classified algorithms into two types. The first uses data mining to investigate different relationships between behaviors and identify abnormal behavior; the second employs a deep CNN that learns from historical data to determine abnormal behavior. On a large database, deep learning algorithms achieved 88 percent accuracy in under 50 s, whereas other solutions achieved less than 81 percent accuracy in under 80 s for pedestrian behavior analysis in smart cities. Shu et al. [90] proposed a graph LSTM-in-LSTM (GLIL) host-parasite architecture for group activity detection, which can be several person LSTM (P-LSTM) or graph LSTM (GLSTM). P-LSTM in a local view and GLSTM in a global view architecture is based on interactions between persons. P-LSTM is integrated and stored into G-LSTM, and residual LSTM learns person-level residual features consisting of temporal features and static features; this was experimented on using a Collective Activity data set (CAD) and Volley-ball data set (VD) dataset to achieve state-of-the-art results as compared to other methods. The summary of literature reviewed related to individual user based HAR is given in Table 6.

#### 4.3.2. Group Activities

Ebrahimpour et al. [91] compared three approaches, namely crowd video analysis, crowd spatio-temporal analysis, and crowd social media analysis, based on different data sources such as sensors and cameras to improve accuracy and quality in smart cities using the Hollywood2 action and Olympic Sports datasets. A convolutional relational machine recognizes group activities with an aggregation component. An activity map based on individual, or group activities was produced by spatial information in the video with multi-stage refinement to reduce errors. The activity map provided better results than other models on volleyball and collective activity datasets [92]. The Multiview-based Parameter Free Framework (MPF) proposed by Q. Wang et al. [93] has two clustering versions based on L1-norm and L2-norm. MPF impacts on designing a descriptor to characterize the structural properties of individuals in the crowd. A self-weighted method makes groups based on features of orientation and context similarity. Q. Wang et al. [93] introduced a framework to automatically detect group numbers in the crowd without any parameter to overcome the limitation of crowd behavior analysis. MPF achieved the best results on real-world crowd video, MSRC-v1, Digits, Caltech101-7, Caltech101-20, and CUHK Crowd Dataset.

H. Ullah et al. [94] proposed a method based on a two-stream CNN architecture. A study combined spatial and temporal networks to handle challenges such as capturing the difference between still frames and motion between frames, as well as the flow field obtained from video through dense flow. The proposed method achieved a 6% improvement on a video dataset over five reference methods. A Coherence Constrained Graph LSTM (CCGLSTM) is based on spatio-temporal context coherence (STCC) and Global context coherence (GCC) constraint with a temporal and spatial gate to control the memory. CCGLSTM is used to recognize group activity at each time stamp by ignoring irrelevant features to overcome the problem of traditional methods. CCGLSTM improved accuracy by 9% and 2% when compared to the other two deep learning models on volleyball and collective activity datasets [95].

Crowd behavior analysis is difficult due to crowd density variation. A two-stream network with heat maps and optical flow information can be used to classify abnormal behavior. Two-stream networks improved accuracy and highlighted the issue of a lack of large-scale datasets [96]. T. Wang et al. [97] proposed an early warning indicator derived from a hidden Markov model (HMM). The proposed method learns HOF orientations in video surveillance based on image descriptors that encode movement information. The classification method for abnormal event detection measures the similarity between normal frames and observed frames. Experimental results showed 97.24% accuracy on the UMN, and PETS datasets.

Simplified Histogram of Oriented Tracklets (sHOT) is a descriptor based on spatio-temporal level and frame level. Spatio-temporal level and frame level orientation and magnitude are extracted in spatio-temporal 3D patches at different levels. The second framework localizes abnormal behavior in video sequences, with 82.2 percent results on UCSD, UMN, and Violence in Crowds [98]. Amraee et al. [99] divided large areas into non-overlapping cells to detect abnormal events accurately. Two distinct one-class SVM models with HOG-LBP and HOF are used to detect abnormal events in crowded scenes. HOG-LBP extracts appearance and HOF motions from the extracted candidate region after removing redundant information. Experimental results showed the best results on UCSD anomaly detection video datasets.

The optical flow method is used to extract values of image pixels as particles from UMN dataset video scenes to find the occurrences of abnormality in the crowd. Their qualities are distributed according to the distance between particles. Then, linear interpolation calculation is applied on a motion foreground to calculate the distance to the camera and determine the timestamp of abnormality [100]. Vahora and Chauhan [101] proposed a contextual relationship-based model that includes context learning from individual activity to group-level activity. Vahora and Chauhan [101] captured human action-pose features of people, then fed these features to RNN to get spatio-temporal group descriptors. The convolutional neural network increased the performance of the proposed model and was evaluated on KTH and Weizman datasets. Experimental results showed that LSTM RNN achieved 82.94% accuracy and GRU RNN achieved 83.45% accuracy. Liu et al. [102] used a predictive neural network to solve the problem of detecting abnormal crowd behavior in public places. Liu et al. [102] defined the degree of anomaly by determining the difference between real frames and predictive frames in the moving object region. The predictive neural network achieved 97.7% accuracy on the UMN dataset when compared to the optical flow method and social force model. An attenuation strategy based on learning rate was used to overcome the Gaussian average model’s slow convergence speed.

Khan [103] presented a method for detecting congestion based on characteristic motion. Motion features are extracted from optical flow and particle advection to show a pattern of increasing trajectory oscillation. The proposed method was evaluated on crowd videos from a self-proposed dataset containing 15 different crowd scenes and demonstrates computational efficiency with an average time of 0.82 s for congestion detection. Results indicated that the method could be used in real time. Enthalpy is used to describe the change in activity as a pedestrian’s motion state changes from normal to panic. Motion information can be obtained with optical flow. Field visualization and texture segmentation methods gain moving regions and are effective. The crowd state is determined using entropy and enthalpy. Experimental results showed 0.97 AUC on the UMN dataset [104].

Gupta et al. [105] proposed a framework CrowdVAS-Net that reduced processing and analysis time for security and crowd management in public places using cameras and YouTube videos. CrowdVAS-Net uses deep CNN for extracting features. These features could be acceleration, velocity, and important features trained with RF classifier. CrowdVAS-Net achieved 77.8% classification accuracy when compared to other state-of-the-art methods on UMN, UCSD, Pets2009, UCF, and self-created video datasets. Direkoglu [106] proposed a new formulation method motion information image for CNN based on optical flow magnitude. The method distinguishes between normal and abnormal behavior in the context of abnormal crowd behavior detection in surveillance videos, caused by natural disasters and violent events. The proposed method produced the best results on the UMN and PETS2009 datasets. Alafif et al. [107] proposed a solution based on optical flow and generative adversarial network (GAN) to address the issue of crowd security. GAN extracts dynamic features and used a transfer learning strategy to detect abnormal behavior. U-Net and Flownet generate normal and abnormal behavior of individuals in the crowd videos with 79.63% accuracy on the abnormal behaviors Hajj dataset. Table 7 presents a summary of the literature related to group-based HAR.

## 5. Data Sources

HAR has become a hot topic in computer vision due to its use in a variety of applications such as healthcare, HCI, security, and surveillance. The type of data generated by various sources, such as videos, images, or signals, has a direct impact on HAR methods. Video is important in HAR because it is used for security, surveillance, and recognizing human activities and behaviors. Vision-based HAR has used a variety of video sources, including CCTV, smartphone cameras, Kinect devices, and social media platforms such as YouTube to detect or predict activities from video streams while Sensor-based HAR is one of the most promising assistive technologies for assisting older people in their daily lives. It focuses on sensor data collected from mobile phone sensors and body wearable sensors such as gyroscopes, accelerometers, Bluetooth, and sound sensors, among others.

**Figure 4:**
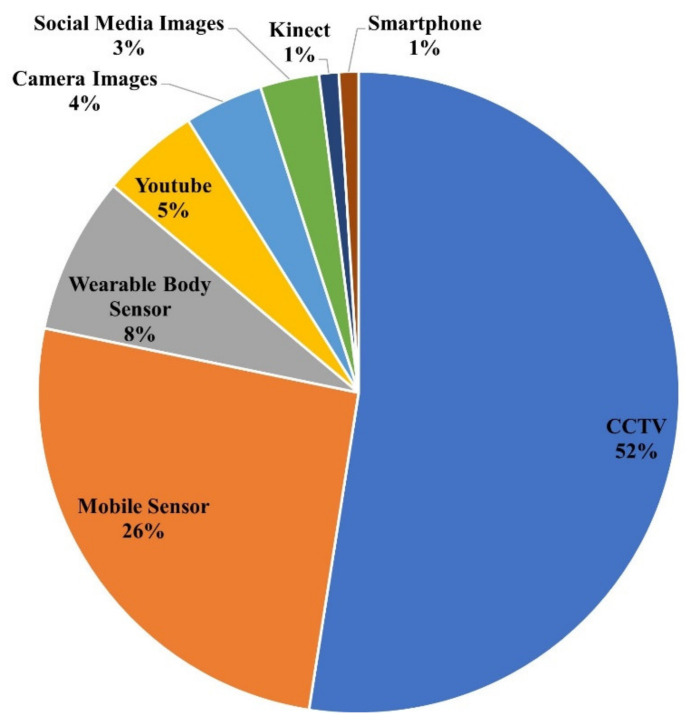
Frequency of data sources used by existing literature on HAR.

This section summarizes the data sources used for HAR. Figure 4 depicts the percentage of each data source, and Table 8 details the data sources used for HAR in the literature. The most common data sources are CCTV cameras (52%) and mobile phone sensors (26%). Other data sources, such as Kinect (1%), smartphone camera (1%), camera images (4%), social media images (3%), wearable body sensors (8%), and YouTube videos (5%), are used less frequently. There are also studies in the literature that did not rely solely on one source of data: [96,103] used CCTV and YouTube, [41] used mobile sensors and wearable body sensors, [82] used mobile camera and mobile sensors.

## 6. Techniques

**Figure 5:**
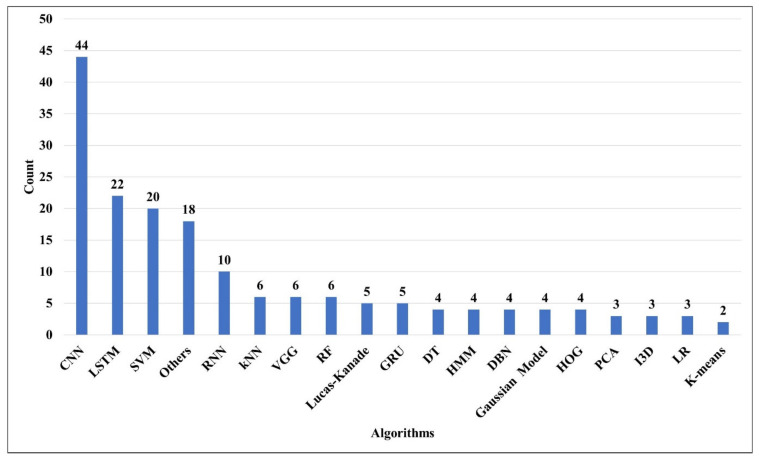
Frequency of techniques/algorithms used in the existing literature on HAR.

Technological advancements have triggered a slew of unprecedented marvels to make our lives easier. Machine learning (ML) is an essential component of recognizing human activity. HAR has used a number of ML algorithms. In ML, there are three types of learning: supervised learning, unsupervised learning, and semi-supervised learning. Some of the most well-known algorithms are as follows: SVM (one class SVM, multi class SVM, Non-Linear SVM, QSVM etc.), RNN (Gated Units, LSTM, BiLSTM, convRNN, DLSTM, BDLSTM, convLSTM, stackedLSTM, P-LSTM, G-LSTM, CCGLSTM, Residual LSTM, etc), NN (CNN, ANN, KNN), CNN (multiheaded CNN, one-dimensional CNN, DCNN, CNN-IMU, RF-DCNN, CNN-BiLSTM, 3D-CNN, R-CNN,2DCNN-LSTM, pre-trained CNN like VGG or YOLO etc) RF, HMM, Naive Bayes (NB), Decision Tree (DT), etc. The techniques used for HAR are summarized in this section. Figure 5 shows the frequency of algorithms used in the literature and Table 9 details algorithms that were used for the task of HAR. The most common techniques are CNN (25%), LSTM (13%), and SVM (12%). Other techniques, such as RNN (6%), kNN(3%), VGG a pre-trained CNN model (3%), Lucas-Kanade(3%), RF (2%), HMM (3%), DT(2%), DBN (3%), Gaussian Model (3%), GRU (3%), HOG (3%), PCA (2%), I3D (2%), K means (1%), LR (1%), are used less frequently. The other category (11%) includes techniques that are not very popular among public users, such as GCC, GAN, CTP, LIC, CRM, CERN, Detectron, FS, GBO, GWO, AGRR.

## 7. Open Challenges and Limitations

The use of HAR in a wide range of application domains provides incomparable benefits, but existing HAR solutions have several limitations and open challenges that must be addressed. This review identifies and highlights data collection, data preprocessing, complex activities, activity misalignment, and hardware limitations.

### 7.1. Data Collection

The process of data collection plays a vital role in data-oriented research. Data collection has a number of issues, according to various studies, including unlabeled datasets, a lack of temporal knowledge, unknown class recognition, and data limitations that must be addressed for activity recognition and prediction. Riaz et al. [67] investigated whether accuracy can be improved by eliminating reliance on data labeling. Alafif et al. [107] aimed to gather more labeled data to improve the accuracy of the classifier. Du et al. [18] used a spatial knowledge-based method and discovered that managing temporal data activities is difficult. Algorithms may struggle to determine the type of activity due to unknown classes of data. Machine learning algorithms have the highest accuracy when trained on known data. Doshi and Yilmaz [71] investigated some cases where the proposed method failed due to unknown classes of data because algorithms require a better understanding of objects and their surroundings. Lee and Shin [84] emphasized the importance of identifying more illegal activities rather than relying on violence and fainting. Zhu et al. [17] acknowledged that current designs are limited to only visible classes and are incapable of recognizing unseen classes, which remains a challenge.

Hsu et al. [77] stated that predicting an individual’s actions is difficult because they vary depending on presence and presentation. Due to the scarcity of large-scale image and video datasets, artificial data generation could be a future project [60]. Nadeem et al. [43] sought a large-scale dataset from hospitals, gyms, and nursery schools to make the proposed model more appropriate and relevant, as models trained on large datasets outperform those trained on small datasets [63]. Köping et al. [13] wished to expand the training dataset in order to improve the classifier’s performance. Saini et al. [31] claimed a lack of data from which to compare their performance and discovered that individual task recording speeds should be matched, otherwise errors occur due to a lack of recorded frames. Lazaridis et al. [96] emphasized the significance of large-scale appropriate datasets. Zhang et al. [100] aimed to gather as many images and videos as possible in order to identify unusual crowd behavior.

### 7.2. Data Preprocessing

Data preprocessing has a significant part in ML and deep learning. After the collection of data, it is very crucial to preprocess data and find some valuable information for HAR. According to different studies “appearance and feature extraction” and “background reduction” are some issues in data preprocessing. Xia and Li [86] focused on the requirement of a feature extractor because vgg16 requires more power. Ullah et al. [94] aimed to build hand-crafted characteristics and merge them with deep learning architecture to increase implementation. Wang et al. [93] focused on the need to develop more cooperative attributes in order to realize crowd actions. Amraee et al. [99] discovered that background images should be calculated after a specific time interval and fed into the proposed method. Jiang et al. [54] aimed to handle instant fluctuations of background in videos.

### 7.3. Hardware and Techniques

Hardware is the backbone of any task especially when a large size of data is involved. Different hardware is used in literature for HAR as we have already discussed such as cameras, smartphones, and different types of sensors. However, according to the literature, there might be some limitations regarding computational cost, hardware problems, and with respect to algorithms. Xia and Li [86] investigated the fact that deep CNN requires a large amount of computation power as a feature extractor and sought to discover a simple algorithm to extract appearance and motion features [44]. As dealing with existing large-scale dataset behavioral analysis takes a significant amount of time, it is planned to investigate other representations of behavioral data [89]. Yoon et al. [44] presented computational complexity as a constraint and proposed designing activity recognition as a multitask learning problem. Bhargava et al. [85] reported that DBN has a longer computation time when detecting anomalies. Ma et al. [45] discovered that recognition results are delayed due to an increase in computation load while determining the start and end time of activity, and this needs to be optimized. Köping et al. [13] investigated the computational problem of nonlinear SVM.

Bhargava et al. [85] present hardware limitations for the neuromorphic chip. Camera placement is very crucial in HAR, and it should be placed in such a way as to cover the complete area. Sometimes the situation needs multiple views and for this purpose, multi-cameras are required to control crowd disasters [105]. Jiang et al. [54] aspire to improve pedestrian recognition from a medium to a long distance with an altitude of fewer than 50 pixels and a breadth of fewer than 20 pixels. Doshi and Yilmaz [71] examined a number of algorithm flaws in which the algorithm missed alarms due to a lack of knowledge about the entity and its surroundings. Higher accuracy can be obtained by investigating the best possible window size, network intensity, and breadth [25]. Belhadi et al. [89] highlighted the need for more different data mining methods and deep learning structural designs to obtain better results. Alafif et al. [107] explored that although the proposed methodology can recognize behaviors, its accuracy could be improved. Lee and Shin [84] highlighted the limitation of algorithms in processing a large amount of data manually. Qi et al. [34] evaluated that DCNN was found to be unsuitable for less labeled data, and the proposed method was compared to other clustering and classification methods. J. Zhang et al. [80] highlighted the need for improvement in the flexibility of the perspective distortion compensation algorithm to obtain accurate weight calculation in a particular situation. X. Zhang et al. [100] aimed to develop objective representation at large-scale and small-scale levels.

### 7.4. Complex Activities Detection

Cooking, reading, and warfare are all examples of complex activities in which some functions are performed using simple actions. According to various sources, complex activities include both complex activities and real-time detection. Helmi et al. [50] mentioned advancements in the developed algorithm to address the problem of concurrent activity datasets. Khan and Ahmad [25] aimed to improve suggested methods for complex actions; additional research is required in this area. Alafif et al. [107] aimed to obtain complex attributes and apply deeper algorithms to large data. According to Ma et al. [45], the proposed algorithm was validated only on wheelchair-related actions, but it can be used for other aspects of activities. In the coming years, real-time biometric user recognition of individuals using cell phone plate forms will be prioritized [26]. According to Mudgal et al. [68] the proposed method could generate real-time alerts from a live streaming camera.

### 7.5. Misalignment of Activities

Manual annotation of data is time-consuming and results in inaccurate labeling and ambiguity in event occurrence timing. A dataset annotation that is incorrectly aligned may reduce HAR accuracy. Furthermore, if the frame length of action is short, repeated instability in prediction can be observed due to frame limit uncertainty [108]. Misalignment occurs when the frame of one action is divided into two or more different frames, resulting in the loss of some useful information during frame segmentation. Furthermore, incorrect action detection is caused by misaligned activities, which reduces the effectiveness of HAR solutions [31]. If only activity end labels are used, the activity may be out of synchronization with the next activity in the frame. Espinilla et al. [33] proposed to process sensor data at regular intervals and determine the best window size for detecting activities based solely on the end time.

## 8. Discussion

Existing HAR literature has addressed a wide range of contexts, including daily living activities, real-time activities, and group-based activities. According to this review, the majority of existing studies focused on static and dynamic living activities, as well as user activities. However, there are very few articles on real-time activity recognition for various purposes such as healthcare, suspicious activities, and surveillance. The small number of studies is due to the complexity of real-time activities, hardware and technical constraints, and limited data availability. In real-time activities, background scenes and context rapidly change in public or crowded areas, necessitating high computation powers to learn of these dynamic changes. Some systems struggled to perform well due to a lack of large amounts of real-time data. Real-time activities are not limited to those described in the literature. Recent technological advancements have encouraged researchers to use HAR to monitor real-time activities of people in pandemics [109,110] and students in online exams [111].

In terms of data collection, depth sensors such as Kinect devices are limited to fall detection and abnormal activity in smart homes due to their limited region of interest. Vision-based HAR provides accurate information only in areas where cameras are present. It does, however, raise privacy concerns because data is constantly being stored, and some people do not want their images or videos to be stored. Furthermore, recognizing multiple actions with a single camera, as well as crowd analysis with a single camera, is extremely difficult. Sensor-based HAR is becoming more popular, and it has both advantages and disadvantages. Some sensors are attached to the human body, while others are attached to various objects such as walls, doors, and so on. When a sensor is placed on the human body, it provides flexible and rich-motion information; however, wearing sensors all the time irritates humans and can cause problems if an individual forgets to wear them. Data loss is a common issue in HAR, but wearable sensors can help with data collection in a variety of real-time environments. Smartphone sensors aided greatly in HAR because almost everyone now owns a smartphone, and its sensors aided in the field of HAR. Devices and sensors are becoming more widely available, and various combinations of these sensors, as well as new devices, could be of interest for HAR.

In the literature, a variety of traditional machine learning techniques, as well as deep learning techniques, have been used for HAR. Researchers propose cutting-edge HAR techniques based on hybrid approaches such as 1D-CNN-LSTM to improve performance. When different algorithms are trained on a small subset of activities, they may experience underfitting, which leads to poor accuracy. In contrast, algorithms trained on the entire dataset face an overfitting problem. Another challenge is the need for specialized hardware such as GPUs for training and testing, which could be addressed by using transfer learning rather than training from scratch. This review cannot claim to include every published article on HAR, but it may be a good starting point for understanding current trends and challenges in this field.

## 9. Conclusions

HAR is now playing a vital role in different domains for surveillance and monitoring. This review aims to categorize existing state-of-the-art literature based on application areas, data sources, techniques, and open research challenges. A total of ninety-five articles related to HAR published since 2018 are selected from different research repositories. It is seen that the majority of existing research (42%) focuses on daily living activities. Furthermore, in daily living activities, dynamic activities such as walking, cooking, washing, reading, etc. are explored much more than static activities such as sitting and standing. User-based (34%) HAR is the second most prominent category explored in the existing literature. It is categorized based on group activities such as crowd behavior and individual user activities such as punching, kicking, and pushing. The real-time activities (24%) explored by existing studies include surveillance, suspicious activities, and healthcare. It is seen that suspicious activities such as theft, shooting, aggressive driving and hiding are explored much more than others. The review of data sources used by existing studies revealed that the majority of existing HAR solutions used vision-based data (70%) from CCTV, YouTube, camera images, social media images, and Kinect devices. Despite the arguments on the advantages of sensor-based data over vision-based data, only a small number of studies (34%) relied on sensor-based data for HAR due to various constraints. It is interesting to see that most of the existing literature used CNN (25%) for HAR, followed by LSTM (13%) and SVM (12%). However, other techniques such as KNN, VGG, etc. are less frequently used. The limitation and open challenges for HAR include data collection, data preprocessing, hardware constraints, the complex nature of activities, and the misalignment of activities. This review will help researchers identify open research challenges associated with existing HAR solutions, as well as provide insights into the current state-of-the-art. Currently, activity recognition is used in a wide range of applications. Future work will consider conducting a review on less explored application domains such as animal activity recognition [112].

## Figures and Tables

**Table 1:** Summary of literature on static activities.

Ref.	Year	Description
[13]	2018	The proposed data integration framework has two components: data collection from various sensors and a codebook-based feature learning approach to encode data into an effective feature vector. Non-Linear SVM used as min method in proposed framework.
[14]	2018	Features were extracted from raw data collected with a smartphone sensor, processed with KPCA and LDA, and trained with DBN for activity recognition.
[15]	2018	PCA is used to reduce dimensionality and extract significant features, which are then compared using a machine learning classifier to raw data and PCA-based features for HAR.
[16]	2019	Introduced SSAL, based on the ST approach to automate and reduce annotation efforts for HAR.
[17]	2019	Proposed a method based on DLSTM and DNN for accurate HAR with smartphone sensors.
[18]	2019	Proposed a three-stage framework for recognizing and forecasting HAR with LSTM: post activity recognition, recognition in progress, and in advance prediction.
[19]	2019	Trained LR, NB, SVM, DT, RF and ANN (vanilla feed-forward) on data collected with low-resolution (4 × 16) thermal sensors.
[20]	2019	Proposed new time and frequency domain features to improve algorithms’ classification accuracy and compare four algorithms in terms of accuracy: ANN, KNN, QSVM, EBT.
[21]	2020	Proposed a pattern-based RCAM for extracting and preserving diverse patterns of activity and solving problem of imbalanced dataset.
[22]	2020	Proposed a method for predicting activities that used a 2-axis accelerometer and MLP, J48, and LR classifiers.
[23]	2020	Investigated that images should be used as HAR sensors rather than accelerometers because they contain more information. Claimed that CNN with images will not burden the modern devices.
[24]	2020	Proposed a hybrid feature selection process in which SFFS extracts features and SVM classifies activities.
[25]	2021	Proposed a one-dimensional CNN framework with three convolutional heads to improve representation ability and automatic feature selection.
[26]	2021	Using CNN and LSTM, a framework CNN-LSTM Model was proposed for multiclass wearable user identification while performing various activities.
[27]	2021	Proposed a CPC framework based on CNN and LSTM for monitoring construction equipment activity.
[30]	2022	Proposed a hybrid model that combines one-dimensional CNN with bidirectional LSTM (1D-CNN-BiLSTM) to recognize individual actions using wearable sensors.
[28]	2022	Aimed to address these issues by employing the GRU network, which collects valuable moments and temporal attention in order to minimize model attributes for HAR in the absence of I.I.D.

**Table 2:** Summary of literature on dynamic activities.

Ref.	Year	Description
[31]	2018	Proposed Coarse-to-Fine framework that uses Microsoft Kinect to capture activity sequences in 3D skeleton form, groups them into two forms, and then classifies them using the BLSTM-NN classifier.
[32]	2018	A deep NN was applied on multichannel time series collected from various body-worn sensors for HAR. Deep architecture CNN-IMU finds basic and complex attributes of human movements and categorize them in actions.
[33]	2018	Proposed online activity recognition with three temporal sub-windows for predicting activity start time based on an activity’s end label and comparing results of NA, SVM, and C4.5 with different changes.
[34]	2019	The proposed FR-DCNN for HAR improves the effectiveness and extends the information collected from the IMU sensor by building a DCNN classifier with a signal processing algorithm and a data compression module.
[35]	2019	Proposed an approach for detecting real-time human activities employing three methods: YOLO object detection, the Kalman Filter, and homography.
[36]	2019	The I3D network included a tracking module, and GNNs are used for actor and object interactions.
[37]	2019	Proposed an ensemble ELM algorithm for classifying daily living activities, which used Gaussian random projection to initialize base input weights.
[38]	2019	Proposed AttnSense with CNN and GRU is for multimodal HAR to capture signal dependencies in spatial and temporal domains.
[39]	2019	Proposed a 3Dimensional deep learning technique that detect multiple HAR using a new data representation.
[40]	2019	Proposed approach has different modules. The first stage generates dense spatiotemporal data using Mask R-CNN, second module has deep 3D-CNN performing classification and localization, then classification using a TRI-3D network.
[41]	2019	Proposed strategy based on 3 classifiers (TF-IDF, TF-IDF + Sigmod, TF-IDF + Tanh) for utilizing statistical data about individual and aggregate activities.
[42]	2019	Demonstrated AdaFrame, which included LSTM to select relevant frames for fast video recognition and time savings.
[45]	2020	In the proposed adaptive time-window-based algorithm, MGD was used to detect signals, define time window size, and then adjust window size to detect activity.
[46]	2020	Implemented CPAM to detect real-time activities in a range calculated by SEP algorithm and reduce energy consumption.
[44]	2020	Proposed a framework for future frame generation, as well as an online temporal action localization solution. Framework contains 4 deep neural network PRs for background reduction, AR for activity type prediction, F2G for future frame generation, and LSTM to recognize action on the basis of input received by AR and PR.
[43]	2020	A HAR framework is proposed that is based on features, quadratic discriminant analysis, and features processed by the maximum entropy Markov model.
[50]	2021	The proposed method improved GBO performance by selecting features and classifying them with SVM using an FS method called GBOGWO.
[48]	2021	The AGRR network has been proposed to solve HOI problems with a large combination space and non-interactive pair domains.
[47]	2021	The ACAR-Net model is proposed to support actor interaction-based indirect relation reasoning.
[49]	2021	In the proposed ASRF framework, an ASB is used to classify video frames, a BRB is used to regress action boundaries, and a loss function is used to smooth action probabilities.
[52]	2022	SVM to identify daily living activities in the proposed system by adjusting the size of the sliding window, reducing features, and implanting inertial and pressure sensors.
[53]	2022	Tried to figure out whether the currently performing action is a continuation of a previously performed activity or is novel in three steps: sensor correlation (SC), temporal correlation (TC), and determination of the activity activating the sensor.

**Table 3:** Summary of literature on surveillance.

Ref.	Year	Description
[54]	2019	Extract static sparse features from each frame by feature pyramid and sparse dynamic features from successive frames to improve feature extraction speed, then combine them in Adaboost classification.
[55]	2019	CNN extracted features from videos after background reduction, fed these features to DDBN, and compared CNN extracted features with labelled video features to classify suspicious activities.
[56]	2020	Identified object movement, performed video synchronization, and ensured proper detail alignment in CCTV videos for traffic and violence monitoring with Lucas–Kanade model.
[58]	2021	Proposed a DPCA-SM framework for detecting suspicious activity in a shopping mall from extracted frames that trained with VGG, along with applications for tracing people’s routes and identifying measures in a store setting.
[57]	2021	Proposed an effective approach to detect and recognize multiple human actions using TDMap HOG by comparing existing HOG and generated HOG using CNN model.
[59]	2021	Proposed an efficient method for automatically detecting abnormal behavior in both indoor and outdoor settings in academics and alerting appropriate authorities. Proposed system process video with VGG and LSTM network differentiates normal and abnormal frames.
[60]	2021	To detect normal and unusual activity in a surveillance system, an SSD algorithm with bounded box explicitly trained with a transfer learning approach DS-GRU is used.
[61]	2022	For dealing with untrimmed multi-scale multi-instance video streams with a wide field of view, a real-time activity detection system based on Argus++ is proposed. Argus++ combined Mask R-CNN and ResNet101.

**Table 4:** Summary of literature on suspicious activities.

Ref.	Year	Description
[62]	2019	PCANet and CNN were used to overcome issues with manual detection of anomalies in videos and false alarms. In video frames, abnormal event is determined with PCA and SVM.
[63]	2020	CCTV footage is fed into a CNN model, which detects shoplifting, robbery, or a break-in in a retail store and immediately alerts the shopkeeper.
[64]	2020	Pretrained CNN model VGG16 was used to obtain features from videos, then a feature classifier LSTM was used to detect normal and abnormal behavior in an academic setting and alert the appropriate authorities.
[65]	2021	The proposed system offered a framework for analyzing video statistics obtained from a CCTV digital camera installed in a specific location.
[66]	2021	Three-dimensional CNN ResNet with spatio-temporal features was used to recognize and detect smoking events.
[67]	2021	A pretrained model was used to estimate human poses, and deep CNN was built to detect anomalies in examination halls.
[68]	2021	The GMM was combined with the UAM to distinguish between normal and abnormal activities such as hitting, slapping, punching, and so on.
[69]	2021	Deep learning was used to detect suspicious activities automatically, saving time and effort spent manually monitoring videos.
[70]	2022	A two-stream neural network was proposed using AIoT to recognize anomalies in Big Video Data. BD-LSTM classified anomaly classes of data stored on cloud. Different modeling choices used by researcher to obtain better results.
[71]	2022	Created a larger benchmark dataset than was previously available and proposed an algorithm to address the problems of continuous learning and few-shot learning. YOLO v4 discovers items from frames and kNN based RNN model avoids catastrophic forgetting from frames.

**Table 5:** Summary of literature on healthcare.

Ref.	Year	Description
[72]	2019	Deep learning was used to create a multi-sensory framework that combined SRU and GRU. SRU is concerned with multimodal input data, whereas GRU is concerned with accuracy issues.
[74]	2020	SDRs were used to create a dataset of radio wave signals, and a RF machine learning model was developed to provide near-real-time classification between sitting and standing.
[73]	2019	Gausian kernel-based PCA gets significant features from sensors data and recognizes activities using Deep CNN.
[75]	2022	“CNN-net”, “CNNLSTM-net”, “ConvLSTM-net”, and “StackedLSTM-net” models based on one dimensional CNN and LSTM stacked predictions and then trained a blender on them for final prediction.
[76]	2022	Used a model based on handcrafted features and RF on data collected with two smartphones.

**Table 6:** Summary of literature on induvial user-based HAR.

Ref.	Year	Description
[77]	2018	SVM and the N-cut algorithm were used to label video segments, and the CRF was used to detect anomalous events.
[78]	2018	A deep convolutional framework was used to develop a unified framework for detecting abnormal behavior with LSTM in RGB images. YOLO was used determine the action of individuals in video frames and then VGG-16 classify them.
[79]	2018	Proposed a HOME FAST spatiotemporal feature extraction approach based on optical flow information to detect anomalies. Proposed approach obtained low-level features with KLT feature extractor and supplied to DCNN for categorization.
[80]	2019	Proposed an algorithm used adaptive transformation to conceal the affected area and the pyramid L-K optical flow method to extract abnormal behavior from videos.
[81]	2019	By combining extracted hidden patterns of text with available metadata, a deep learning architecture RNN was proposed to detect abusive behavioral norms.
[10]	2019	SVM was used to determine abnormal behavior using extracted feature vectors and vector trajectories from the computed optical flow field of determined joint points with LK method.
[82]	2019	The proposed LSTM-FCN detects aggressive driving sessions as time series classification to solve the problem of driver behavior.
[83]	2019	A method that combined CNN with HOF and HOG was proposed to detect anomalies in surveillance video frames.
[84]	2020	A deep learning model was used to detect abnormal behavior in videos automatically, and experiments with 2D CNN-LSTM, 3D CNN, and I3D models were conducted.
[85]	2020	Propose to do instance segmentation in video bytes and predicting the actions with the help of DBN based on RBM. Aimed to present an implementation of an algorithm that can depict anomalies in real time video feed.
[86]	2021	Proposed a method for detecting abnormal behavior that is both accurate and effective. VGG16 network transferred to full CNN to extract features. Then LSTM is used for prediction at that moment.
[87]	2021	Proposed a method in the ABAW competition that used a pre-trained JAA model and AU local features.
[88]	2021	Proposed a strategy for recognizing and detecting anomalies in human actions and extracting effective features using a CPRTSA based Deep Maxout Network.
[89]	2021	The algorithm was classified into two types. The first employs data mining and knowledge discovery, whereas the second employs deep CNN to detect collective abnormal behavior. Researcher planned variation of DBSCAN, kNN feature selection, and ensemble learning for behavior identification.
[90]	2021	Residual LSTM was introduced to learn static and temporal person-level residual features, and GLIL was proposed to model person-level and group-level activity for group activity recognition.

**Table 7:** Summary of literature on group-based HAR.

Ref.	Year	Description
[96]	2018	A two-stream convolutional network with density heat-maps and optical flow information was proposed to classify abnormal crowd behavior and generate a large-scale video dataset. To prevent long-term dependency, they used LSTM.
[97]	2018	For abnormal event detection in surveillance videos, an algorithm based on image descriptors derived from the HMM that used HOFO as feature extractor and a classification method is proposed.
[98]	2018	The proposed descriptor is based on spatiotemporal 3D patches and can be used in conjunction with sHOT to detect abnormal behavior. Then one class SVM classifies behaviors.
[99]	2018	HOG-LBP and HOF were calculated from extracted candidate regions and passed to two distinct one-class SVM models to detect abnormal events after redundant information was removed.
[100]	2018	Particle velocities are extracted using the optical flow method, and motion foreground is extracted using the crowded motion segmentation method. The distance to the camera is calculated using linear interpolation, and crowd behavior is analysed using the contrast of three descriptors.
[91]	2019	Reviewed crowd analysis fundamentals and three main approaches: crowd video analysis, crowd spatiotemporal analysis, and social media analysis.
[92]	2019	Presented a deep CRM component that learns to generate activity maps, a multi-stage refinement component that reduces incorrect activity map predictions, and an aggregation component that recognizes group activities based on refined data.
[101]	2019	Presented a contextual relationship-based learning model that uses a deep NN to recognize a group of people’s activities in a video sequence. Action-poses are classified with pre-trained CNN, then passed to RNN and GRU.
[102]	2019	A Gaussian average model was proposed to overcome the disadvantage of slow convergence speed, and a predictive neural network was used to detect abnormal behavior by determining the difference between predictive and real frames.
[103]	2019	Extracted optical flow motion features, generated a trajectory oscillation pattern, and proposed a method for detecting crowd congestion.
[104]	2019	A method for detecting crowd panic states based on entropy and enthalpy was proposed, with enthalpy describing the system’s state and entropy measuring the degree of disorder in the system. Crowded movement area represented in the form of text with LIC.
[105]	2019	The CrowdVAS-Net framework is proposed, which extracts features from videos using DCNN and trains these features with a RF classifier to differentiate between normal and abnormal behavior.
[93]	2020	The proposed MPF framework is built on the L1 and L2 norms. Descriptor of structure context for self-weighted structural properties Framework for group detection and multiview feature point clustering.
[106]	2020	MII is generated from frames based on optical flow and angle difference and used to train CNN, provide visual appearance and distinguish between normal and unusual crowd motion with one class SVM.
[94]	2021	The proposed method employs a two-stream convolutional architecture to obtain the motion field from video using dense optical flow and to solve the problem of capturing information from still frames.
[107]	2021	Extracted dynamic features based on optical flow and used an optical flow framework with U-Net and Flownet based on GAN and transfer learning to distinguish between normal and abnormal crowd behavior.
[95]	2022	CCGLSTM with STCC and GCC is proposed to recognize group activity and build a spatial and temporal gate to control memory and capture relevant motion for group activity recognition.

**Table 8:** Vision-based and Sensor-based data sources used in the literature.

Ref.	Vision-Based						Sensor-Based	
	CCTV	Kinect Device	YouTube	Smart Phone	Camera Images	Social Media Images	Mobile Sensor	Wearable Device Sensor
[97]	✔							
[98]	✔							
[13]							✔	
[33]							✔	
[31]		✔						
[14]							✔	
[77]	✔							
[96]	✔		✔					
[99]	✔							
[15]							✔	
[65]	✔							
[54]	✔							
[32]	✔							
[78]	✔							
[79]	✔							
[101]	✔							
[80]	✔							
[100]	✔							
[34]							✔	
[72]								✔
[18]								✔
[35]	✔							
[16]							✔	
[36]	✔							
[81]						✔		
[20]							✔	
[39]	✔							
[37]							✔	
[17]							✔	
[40]	✔							
[10]	✔							
[102]	✔							
[41]							✔	✔
[73]								✔
[42]			✔					
[82]				✔			✔	
[62]	✔							
[38]							✔	
[91]	✔							
[103]	✔		✔					
[92]	✔							
[104]	✔							
[105]	✔		✔					
[83]	✔							
[55]	✔							
[44]	✔							
[84]	✔							
[85]	✔							
[21]							✔	
[106]	✔							
[43]						✔		
[45]							✔	
[63]						✔		
[74]							✔	
[22]							✔	
[23]					✔			
[56]	✔							
[64]	✔							
[93]	✔							
[24]							✔	
[46]								✔
[48]					✔			
[57]	✔							
[86]	✔							
[50]							✔	
[87]	✔							
[47]	✔							
[49]	✔							
[67]	✔							
[25]							✔	
[26]							✔	
[88]	✔							
[27]							✔	
[89]					✔			
[58]	✔							
[59]	✔							
[107]	✔							
[90]	✔							
[94]	✔							
[68]	✔							
[69]	✔							
[60]	✔							
[66]	✔							
[61]	✔							
[70]	✔							
[71]	✔							
[51]							✔	
[95]			✔					
[76]							✔	
[53]							✔	
[30]								✔
[19]	✔							
[52]								✔
[75]							✔	✔
[28]							✔	

**Table 9:** Techniques/algorithms used in the literature.

Ref.	SVM	KNN	RF	DT	CNN	RNN	LSTM	HMM	PCA	DBN	K-Means	VGG	Lucas-Kanade	Gaussian Model	I3D	LR	GRU	HOG	Others
[97]								✔											
[98]	✔																		✔
[13]	✔																		
[33]	✔			✔															
[31]							✔	✔											
[14]	✔				✔					✔									
[77]	✔																		
[96]					✔		✔												
[99]	✔																	✔	
[15]	✔			✔	✔				✔										
[65]		✔																	
[54]																		✔	
[32]					✔														
[78]					✔		✔					✔							
[79]					✔								✔						
[101]					✔	✔	✔										✔		
[80]											✔		✔						
[100]																			✔
[34]					✔														
[72]						✔											✔		
[18]							✔												
[35]																			✔
[16]																			✔
[36]															✔				
[81]						✔													
[20]	✔	✔			✔														
[39]					✔														
[37]																			✔
[17]	✔	✔					✔									✔			
[40]					✔	✔									✔				
[10]	✔												✔						
[102]														✔					
[41]																			✔
[73]					✔				✔										
[42]							✔												
[82]			✔		✔		✔												
[62]	✔				✔				✔										
[38]	✔		✔		✔		✔										✔		
[95]							✔												
[91]	✔				✔	✔	✔				✔		✔						
[103]																			✔
[92]					✔														
[104]																			✔
[105]			✔		✔														
[83]					✔													✔	
[55]					✔					✔									
[44]					✔		✔												
[84]					✔		✔								✔				
[85]										✔									
[21]						✔													
[106]	✔				✔														
[43]								✔											
[45]														✔					
[63]					✔														
[74]	✔	✔	✔		✔														
[22]				✔												✔			
[23]					✔														
[56]													✔						
[64]					✔	✔						✔							
[93]																			✔
[24]	✔																		
[46]																			✔
[57]					✔													✔	
[86]					✔		✔					✔							
[50]	✔																		
[87]																			✔
[48]																			✔
[47]																			✔
[49]																			✔
[67]					✔														
[25]					✔														
[26]					✔		✔												
[88]																			
[27]					✔		✔												
[89]		✔																	
[58]	✔				✔			✔		✔									
[59]					✔		✔					✔							
[107]					✔														
[90]							✔					✔							
[94]					✔														
[51]																			✔
[68]														✔					
[69]					✔									✔					
[60]																	✔		
[66]					✔														
[61]						✔													
[70]					✔	✔	✔					✔							
[71]		✔				✔	✔												
[75]					✔		✔												
[28]																	✔		
[53]																			✔
[52]	✔																		
[30]					✔		✔												
[76]																			✔
[19]	✔		✔	✔												✔			
